# A method for positive forensic identification of samples from extremely low-coverage sequence data

**DOI:** 10.1186/s12864-015-2241-6

**Published:** 2015-12-07

**Authors:** Samuel H. Vohr, Carlos Fernando Buen Abad Najar, Beth Shapiro, Richard E. Green

**Affiliations:** Department of Biomolecular Engineering, University of California, Santa Cruz, 1156 High St, Santa Cruz, CA 95064 USA; Facultad de Ciencias, Universidad Nacional Autónoma de México, Av. Universidad 3000 Circuito Exterior S/N, Delegación Coyoacán, Ciudad Universitaria, C.P. 04510 Ciudad de México, D.F. México; Department of Ecology and Evolutionary Biology, University of California, Santa Cruz, 1156 High St, Santa Cruz, CA 95064 USA

**Keywords:** Forensics, Ancient DNA, Genomics

## Abstract

**Background:**

Determining whether two DNA samples originate from the same individual is difficult when the amount of retrievable DNA is limited. This is often the case for ancient, historic, and forensic samples. The most widely used approaches rely on amplification of a defined panel of multi-allelic markers and comparison to similar data from other samples. When the amount retrievable DNA is low these approaches fail.

**Results:**

We describe a new method for assessing whether shotgun DNA sequence data from two samples are consistent with originating from the same or different individuals. Our approach makes use of the large catalogs of single nucleotide polymorphism (SNP) markers to maximize the chances of observing potentially discriminating alleles. We further reduce the amount of data required by taking advantage of patterns of linkage disequilibrium modeled by a reference panel of haplotypes to indirectly compare observations at pairs of linked SNPs. Using both coalescent simulations and real sequencing data from modern and ancient sources, we show that this approach is robust with respect to the reference panel and has power to detect positive identity from DNA libraries with less than 1 % random and non-overlapping genome coverage in each sample.

**Conclusion:**

We present a powerful new approach that can determine whether DNA from two samples originated from the same individual even when only minute quantities of DNA are recoverable from each.

**Electronic supplementary material:**

The online version of this article (doi:10.1186/s12864-015-2241-6) contains supplementary material, which is available to authorized users.

## Background

Recent advances in high-throughput sequencing now allow for small DNA fragments to be extracted and sequenced from ancient or heavily degraded samples [1]. DNA is now routinely extracted from bones, teeth, and hair from individuals that lived hundreds to tens of thousands of years ago and from small and poorly preserved forensic samples. Deep sequencing of well-preserved samples can produce many-fold coverage of the complete nuclear genome of an individual [2–4], but more often samples yield only small amounts of endogenous DNA, i.e., less than one-fold genome coverage [5–7]. This is due to several factors. First, the amount of intact DNA varies greatly between samples. Also, in many samples endogenous DNA makes up only a small fraction of the DNA that can be extracted and sequenced. Deep sequencing and capture techniques [8–11] can be applied to recover more human DNA from each sample but, ultimately, the total amount of useful human DNA sequence is limited by the number of intact human DNA fragments in the sample. In many cases, no enrichment or deep sequencing strategy will increase the amount of data collected.

A common task in forensics and in the analysis of archeological and historical samples is to determine whether two samples originated from the same individual by comparing DNA extracted from each. The most widely adopted approach in forensics is to compare genotype information at a defined set of multi-allelic short tandem repeat (STR) markers [12, 13]. These genotype data can be generated by PCR amplification using primer pairs flanking the markers followed by capillary electrophoresis or, more recently, from deep sequencing of these ampliconic products [14, 15]. This approach is fully effective only when there are amplifiable template molecules for each of the two alleles of each marker. Thus, it is not uncommon for this approach to fail or generate only a partial DNA profile from minute or poorly preserved samples.

The advent of high-throughput sequencing is enabling new approaches to compare DNA between samples [16, 17]. For example, STR profiles can be genotyped from shotgun data by altering the search strategy used to map shotgun reads to a reference genome [18, 19]. These STR data can then be compared to available data generated using more traditional approaches. More recently, some researchers have begun to use single nucleotide polymorphism (SNP) markers, which are more easily typed by whole genome shotgun sequencing [20, 21]. As of July 2015, the Single Nucleotide Polymorphism Database (dbSNP) contains records for 149,735,377 SNP clusters in the human genome [22]. Despite the vast number of SNPs, SNP-based comparison methods typically employ no more than a few hundred markers, in part to avoid the effects of genetic linkage, as this would render each comparison non-independent during analysis. Nevertheless, this enormous catalog is potentially a powerful resource for sample comparison and identification, as it provides much higher probability that genotype data at overlapping markers can be learned.

Here we describe a new approach for comparing very sparse DNA sequence data from two samples that differs from existing methods in two important ways. First, we draw on large catalogs of SNP markers with the expectation that only a small fraction of these will be observed in a sample. Second, we completely avoid direct comparison of alleles between samples. In comparisons of poorly preserved samples, direct comparison is difficult since few or zero SNP positions will be observed in both samples and full genotypes are not recoverable. In addition, multiple observations of the same SNP position may be the result of incorrectly mapped reads from similar sequences in the genome. To address this, our method does not require genetic observations at the same loci. Instead, we compare pairs of observations at linked markers to assess whether the observations more likely originated from a single individual or from independent individuals. This is made possible by the large and growing catalog of known segregating genetic variation in humans and the known patterns of linkage present in human populations. Using this approach we show that it is possible to reliably determine if two samples originated from the same individual when the data available are random, non-overlapping shotgun sequence data representing less than 1 % of the genome in both samples.

## Methods

Two loci are in linkage disequilibrium (LD) if their alleles are not randomly associated [23]. As the name implies, LD can occur because alleles that are physically linked and nearby on chromosomes are often co-inherited. This non-random association implies that observing the allelic state of one locus provides some information about the state of the other. Our approach is based on the idea that observations of alleles made in one sample consistently provide information about the allelic state in another sample via LD if and only if the samples are from the same individual (or from a genetically identical individual, i.e., a monozygotic twin). On the other hand, when comparing data from samples from unrelated individuals, each sample provides no predictive information about alleles in the other sample.

**Fig. 1:**
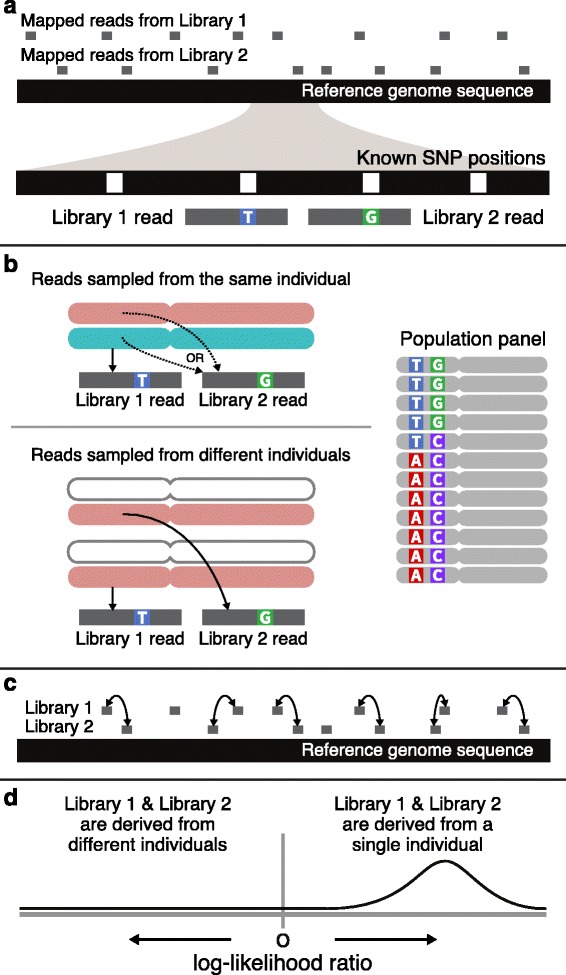
Overview of the method. **a** Sequence data from two extremely low-coverage shotgun libraries are mapped to a reference sequence. Reads that overlap known single nucleotide polymorphimic (SNP) sites are used to observe a single base for that position. The majority of SNP positions have no observations. Pairs of closely linked SNPs with observed bases are identified. ***b*** For each SNP pair, we calculate the probability of the observations under two models. The first model represents the case where the two reads orginated from a single diploid individual (*top*). In this model there is an equal chance that the two reads were drawn from the same chromosome or different chromosomes. This model takes the haplotype frequency as well as allele frequency into account. The second model represents the case where the two reads originated on different chromosomes (*bottom*). The probability of observing the two bases is the product of the allele frequencies. A reference panel of phased haplotypes is used to model the allele and haplotype frequencies for the population from which the samples were drawn. These probablilies are compared as a log-likelihood ratio. **c** Comparisons are made for any pair of SNPs occuring within a specified distance along a chromosome. **d** log-likelihood values are aggregated by sampling pairs from windows across the genome to avoid confounding effects from linkage. This sampling step is repeated in a bootstrapping approach to build an empirical distribution of the genome-wide log-likelihood ratio. Positive values indicate that the single, diploid individual model is favored while negative values indicate that the two samples are from independent individuals

Consider a pair of SNP loci in physical proximity on the same chromosome in linkage disequilibrium (Fig. 1). Alleles (bases) are observed for each of these two positions from independent shotgun reads that overlap these SNP positions in two independent sequence samples (libraries). We compare the probabilities of observing these two bases under two models. The first model represents the case where the two reads, and thus the two alleles they contain, were drawn from independent chromosomes. Under this model, the probability of the observation is simply the product of the two allele frequencies in the population. The second model represents the case where the two bases were drawn from the same diploid individual. Under this model there is an equal chance that this second read was drawn from the same chromosome as the first read or the other chromosome in that individual. In the case that the read derives from the other chromosome, its allelic state is also not informed by the first read. When drawn from the same chromosome as the first read, however, the probability of observing the alleles seen on the first and second reads is given by the LD between these two alleles, i.e. the haplotype frequency of these two alleles. The two models can be used to evaluate the probability of any pair of allelic observations from two libraries to determine if it is more likely that the two reads originated from one individual or not.

To estimate these probabilities, we model allele and haplotype frequencies in an approach similar to the one employed by Rodriguez et al. in the identity by descent detection method Parente2 [24]. Allele and haplotype frequencies are explicitly modeled using a reference panel of phased haplotypes. Since this reference panel is used to discover segregating polymorphic positions, their allele frequencies, and the patterns of linkage of these sites, our approach benefits when the population reference panel and samples are drawn from genetically similar populations. In practice, these panels can be constructed from statistically phased genotypes from surveys of human genetic diversity, such as the 1000 Genomes Project [25].

For every SNP pair within a specified distance, the probabilities under the single individual and two individual models are calculated and compared as a log-likelihood ratio (LLR). A positive value indicates that the probability of observing these bases under the single individual model is higher than that under the two individual model. A negative value indicates that the data are more likely to derive from two different individuals. A single comparison of a pair of SNPs offers little information to distinguish between these two models on its own. However, the two models can become more distinguishable by combining observations from many pairs of SNPs across the genome. To achieve this, we aggregate log-likelihood ratios for pairs of SNPs by sampling pairs from sliding windows across each chromosome. We chose a wide window size (500 kb) and pick a suitable pair of alleles within each window. The human genome, at roughly three gigabases, contains approximately 6000 independent windows for these comparisons. This approach allows us to treat each window as an independent assessment of the two models. Then, we generate an overall LLR by summing the values across all windows where a suitable comparison can be made. Note that in any particular window, it is only necessary that at least one read in each library contains allelic information for at least one SNP position. As described below, this allows analysis of datasets of extremely low overall coverage.

We repeat this process in a bootstrapping approach, selecting random pairs of alleles across each window, to construct an empirical distribution of the genome-wide aggregated log-likelihood ratio. From this distribution, we can assess the mean and variance of the LLR from the underlying data.

As described here, this method is applied to sets of base observations made from shotgun data from two samples. In addition to this, the method can be applied to a set of base observations made from a single sample. When done in this way, the analysis tests if pairs of observations from a single sample are internally consistent. In this case, positive LLR values indicate that the sample data is consistent with a single, diploid individual. Negative LLR values or values not significantly different from zero indicate that the sample is a mixture of fragments from more than one individual or that the reference panel is too distantly related from the sample individual.

## Results

### Results from simulations

**Fig. 2:**
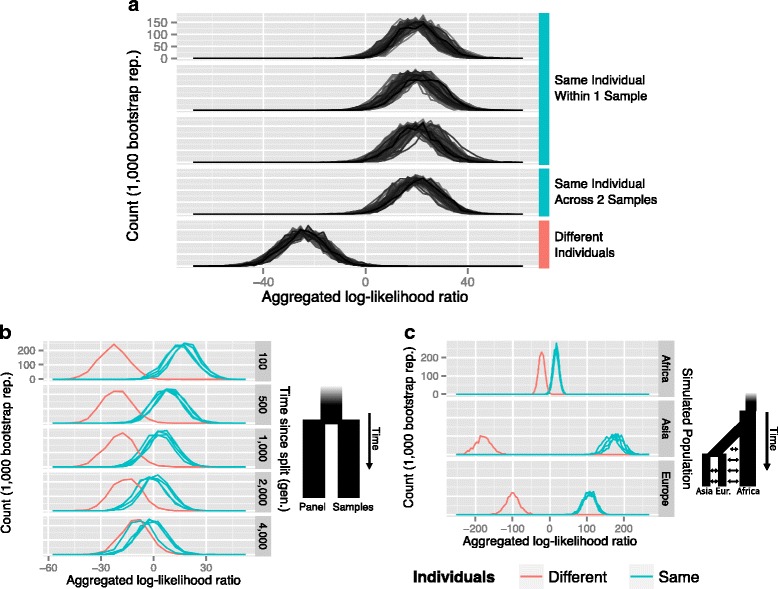
Summary of results from coalescent simulations. **a** Results from 100 replicates of comparisons between three low-coverage subsamples from two simulated diploid individuals with a reference panel of haplotypes drawn from the same population. The first three panels report the results of comparisons made with alleles observed within the same sample. The aggregated log-likelihood ratio values are largely positive, indicating that the observations are consistent with originating from a single diploid individual. Similarly, the fourth panel shows positive values for the comparison made between two independent subsamplings of the same diploid individual. In the last panel, comparisons of allele observations made from two independent individuals produce negative aggregated log-likelihood values, indicating the two samples are from different individuals. **b** A variation of the comparisons made in Panel **a** where samples from diploid individuals are compared using a panel of reference haplotypes from a related, but diverged population (see diagram). Each panel shows the results of five comparisons; four made between samples from the same individual (*blue*) and one made between different individuals (*red*). From these results, a closely related population retains power to differentiate between one or two individuals over many generations. As the number of generations since the population split increases, the power of the related population to model of the population from which the samples were drawn diminishes. **c** Results from a simulated model of recent human population history (see diagram and Additional file 1). In each subpopulation, comparisons between samples from diploid individuals using a panel of haplotypes and differentiate between the four comparisons made against the same individual and the one comparison made between different individuals. Increased linkage disequilibrium in subpopulations that have undergone recent population bottlenecks (Europe and Asia) increases the power of the method to differentiate between one and two individuals

To examine the feasibility and power of this method, we simulated sets of single allele observations from diploid individuals along with panels of reference haplotypes using coalescent simulations and tested our method under various demographic scenarios, free from observation errors. We first simulated observations under a simple demographic model of a single population of constant size (N_e_ = 10,000). Since this model lacks population bottlenecks that increase genome-wide LD, we consider this to be a conservative model. In each round of simulation, we generated two diploid individuals by drawing two haplotypes each from the simulations results. Alleles from these individuals were sampled at a rate of 0.02 to produce sets of allelic observations similar to what could be achieved in genome sequencing from libraries that represent 0.02 fold genome coverage. At this level of coverage, traditional methods like STR typing could be expected to fail. The remaining haplotypes, i.e., those not drawn for comparison, were used as the reference panel to model allele and haplotype frequencies. From 100 rounds of simulation, we found our method can consistently distinguish between two single allele observation sets that originate from the same or different individuals (Fig. 2a) when the reference panel and diploid individuals are drawn from the same population. Within-sample comparisons produced results that were nearly identical to across sample comparisons from the same simulated individual. In addition to this, we found that simulated parent-child and sibling-sibling comparisons produce intermediate results centered around 0 (see Additional file 1).

In many cases, an ideal reference panel may not be available to describe the variation present in the population from which the samples originated. However, a related population may provide sufficiently similar allele frequencies and patterns of LD to power the approach. To test this, we extended our simulations to model a population split, producing two equally sized populations with no subsequent migration, for various times in the past. We drew the haplotypes for the diploid individuals from one population and the haplotypes for the reference panel from the other. In this way, genetic drift and independent recombination histories will alter allele frequencies and LD in a way that reduces power in our method. Nevertheless, we found that a reference panel separated by hundreds of generations still provides information to differentiate between samples that come from one individual or two (Fig. 2b). However, discrimination becomes increasingly difficult as the number of generations since the split of these populations increases.

To test our method on a realistic demographic model for humans, we simulated a demographic history based on parameters inferred from SNP frequency data [26]. We simulated diploid individuals and reference population panels for three subpopulations representing populations in Africa, East Asia and Europe. Importantly, this model features historical bottlenecks that increase and restructure LD in the affected subpopulations. Comparisons made within each subpopulation can differentiate between samples that come from one or two individuals (Fig. 2c). We also noted that the separation between single individual and two individual comparisons varied between populations due to their distinct demographic histories. The subpopulations that have undergone more recent bottlenecks (Europe and Asia) and thus have higher LD consequently have much clearer separation between models. Strikingly, the subpopulation with the greatest separation (Asia) is the one that has undergone the strongest recent bottleneck. In contrast, the simulated African subpopulation, with the largest N_e_ and thus the least LD, has the least power to distinguish individuals. Notably, comparisons where the sample data and reference variation were drawn from different populations produced results that were more difficult to interpret (see Additional file 1). These observations highlight the importance of choosing a suitable reference panel.

### Results from shotgun sequencing data

To test our method on extremely low-coverage shotgun sequencing data, we obtained read data from a European male (NA12891) and a European female (NA12892) sequenced as part of Illumina’s Platinum Genomes [27]. We sampled 3200, 32,000, 160,000, 320,000 and 1,600,000 reads without replacement to approximate 0.01, 0.1, 0.5, 1.0, and 5.0 % fold coverage of the nuclear genome to generate two sets at each coverage level for both individuals. Only the forward reads (101 bp) from each pair were used, to approximate the effect of having short fragments that must be reliably mapped without mated reads. Reads were mapped to the human reference genome (hg19) using BWA [28]. Only bi-allelic single base substitutions were included in the panels. Base observations were made from the mapped reads using samtools mpileup after coverage, map quality and base quality filtering [29]. We constructed reference population panels using the statistically phased haplotypes from the 1000 Genomes Project phase one data [25].

**Fig. 3:**
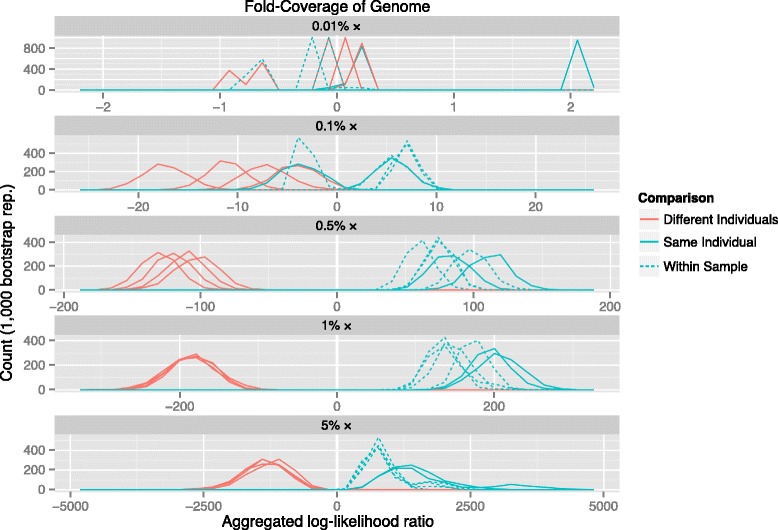
Results from comparisons made with single base observations from extremely low-coverage sequencing data. Each panel shows the results of 10 comparisons made between four low-coverage subsamples of reads sequenced from two human individuals (NA12891 and NA12892 from the CEU pedigree) using a reference panel of haplotypes from the 1000 Genomes CEU subpopulation. Comparisons made from samples from the same individual are shown in blue, with comparisons made within the sample shown as a dashed line. Comparisons made from samples from different individuals are show in red. At the extremely low coverage of 0.01 % fold, very few usable pairs of SNPs can be found. As coverage increases above 0.1 % fold, the two groups of comparisons are easily separable. Comparisons made within a sample have less extreme LLR values as they were made with less read coverage than across-sample comparisons

We compared each sample to itself and to the other three at the same coverage level using a reference panel of 170 haplotypes from the CEU population (4,444,573 SNPs in total). Pairs of SNPs were separated by 1 to 50 Kb and aggregated LLRs were calculated by sampling pairs in 500 Kb windows (Fig. 3). At the lowest coverage level, 0.01 %, comparisons were not possible as we found very few pairs in close proximity due to the low observation density. At 0.1 % x coverage, the model distributions can be seen separating away from 0, with the comparisons made between the same individuals and comparisons between different individuals trending towards positive and negative respectively. With coverage levels 0.5 % and higher, comparisons between the same and different individuals are distinct and easily differentiable. We found similar patterns when using reference panels from other European populations (see Additional file 1).

### Results from ancient DNA sequencing data

Finally, to test our method on genuine ancient DNA sequencing data, we selected 12 samples from 11 Bronze Age Eurasian humans sequenced by Allentoft et al. [7]. After independent DNA extraction and sequencing, the authors discovered that two of these samples (RISE507 and RISE508) were from the same individual through genetic comparison and consultation of the museum records. Sequencing of DNA extracted from these tooth samples produced 0.13 and 0.26 fold coverage of the genome. In addition to these two, we randomly selected 10 other samples sequenced from bones and teeth with similar coverage levels from the data set. We applied our method to all 78 pairwise comparisons between the 12 samples using the same parameters and CEU reference panel of present day humans described in the previous section. We limited our comparison to SNPs that represented transversion mutations to avoid erroneous observations caused by the C to T and G to A substitutions associated with ancient DNA damage [30].

**Fig. 4:**
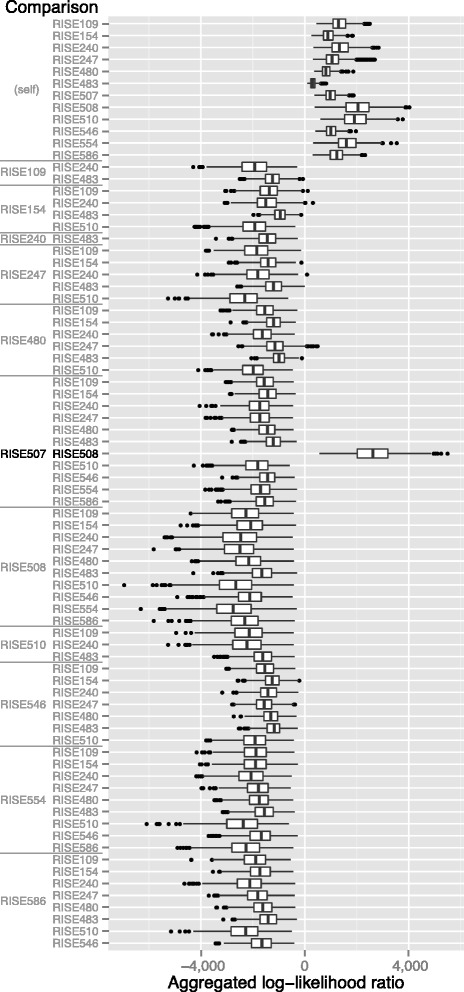
Results from comparisons of low-coverage ancient DNA sequencing. Low-coverage (0.1–0.26 fold) sequence data from 12 ancient DNA samples from 11 Bronze Age Eurasian individuals were compared to each other using a panel of haplotypes from the 1000 Genomes CEU population. All comparisons made within a sample produce positive aggregated log-likelihood ratio values indicating that they are consistent with originating in a single diploid individual. Comparisons made between samples produce negative aggregated log-likelihood values, confirming that the two samples are from are from different individuals. The exception to this is the comparison between samples RISE507 and RISE508, the two samples reportedly made from the same individual, indicating that they are consistent with originating in the same individual

All 12 within-sample comparisons produced positive aggregated LLR distributions (Fig. 4) indicating that each sample is consistent with originating in a single diploid individual. In contrast, comparisons made between data from different samples are strongly negative, indicating that the two samples most likely originated in different individuals. The exception to this is the comparison between samples RISE507 and RISE508 where the aggregated LLR distribution is strongly positive, correctly indicating that they originated from the same individual. Taken along with mitochondrial result from the original work, this result provides confirmation that the two samples are indeed from the same individual. Variation between results appears to be primarily due to differences in the coverage of the genome in each comparison (see Additional file 1).

## Discussion

We present a method for comparing sparse shotgun read data from two samples to determine whether the two samples originated from the same individual. Our method assumes that observations of alleles from one sample provide information via LD about the allelic state in another sample if both samples derive from the same individual. In addition, because ancestry within an individual and patterns of LD in human populations are local within the genome, we provide quantitative assessment of model fit by considering genomic regions individually and aggregating information across the genome. In this way, our approach provides a robust statistical assessment of whether two samples derive from the same individual or from more than one individual.

Our approach performs well even with as little as 0.01 fold genomic coverage each from the two samples. The quantity of sample necessary to achieve this level of coverage will vary from sample to sample but is attainable in many cases where traditional forensics assays would fail. This amount of genomic data is equivalent to 30 femtograms of human DNA recovered and sequenced. While the efficiency of DNA recovery and library generation is not perfect [31, 32] and poorly preserved samples are often mixed with environmental DNA [1], our approach should allow useful comparative analysis for samples that were heretofore intractable for forensic analysis.

Central to our approach is the use of a large panel of phased SNP haplotypes from a reference population. For optimal discriminatory power, the reference should be of the same or a closely related population. To select the most appropriate reference panel, one might perform principal components analysis or some other classifier on the sparse data set prior to analysis. However, because haplotype variation across the human genome is often shared among human populations [33, 34], our approach has power even in cases of a poorly chosen reference panel. In many cases, a suitable reference population may not be available, especially for individuals who draw ancestry from multiple distinct populations. In these cases, comparing results from within-sample comparisons using different haplotype panels may be useful in identifying the most appropriate panel for comparisons between samples. More work will be needed to understand how admixture affects the results of comparisons to make the method fully applicable to present-day individuals and populations.

As described, our approach is designed to test the relative fit of two models: that the samples originated from the same individual or that the samples are derived from unrelated individuals from the same population. If the two individuals are unrelated and from different populations, then the structure of the test would predict even more strongly that they are two unrelated individuals. One obvious extension of this approach is to test other models of relatedness. For example, our method as described has some power to detect parent-child and sibling-sibling relationships (see Additional file 1). Our approach could be further extended to detect more distant relationships between individuals. Another useful application of this method is to compare pairs of observations made from within a single sample. Using this approach, it is possible to assess the relatedness of the sample to *itself*, which is functionally equivalent to a test for contamination or a mixture of samples. Further work will be required to explore the limits of sensitivity for contamination, the impact of complex mixtures in samples, and the limits of sensitivity in real world forensics, historical, and ancient DNA.

## Conclusion

Through the method described here, we demonstrate that by using a large collection of SNP markers and patterns of linkage disequilibrium modeled by a panel of haplotypes, sets of non-overlapping, low-coverage sequencing data can be compared to determine if the two samples originated from the same individual.

### Additional methods

#### Algorithm

We begin with a SNP reference panel of haplotypes from a population chosen based on a priori knowledge or previous analyses to best represent the population from which the samples originated. For each sample, we examine reads that overlap SNP positions from the panel to identify the base at that position. Bases that do not match one of the two alleles from the reference panel are discarded. Observations for positions where multiple reads map are omitted. The majority of positions will have no observation.

Next, we find all pairs of SNPs between the two samples within a specified distance on the chromosome. A minimal distance is also enforced to ensure that the two base observations are never made from the same fragment in within-library comparisons. For each pair of base observations, denoted as *A* and *B*, we calculate the probabilities of that observation under two models. The first represents the probability of observing this combination of bases when the observations were made from independent chromosomes, i.e. two unrelated individuals. In this case, the two observations are independent and based solely on the frequencies of each allele in the population:

$$ {P}_2\left(A\wedge B\right)=f(A)f(B) $$

Where *f*(*A*) and *f*(*B*) are the frequencies of alleles *A* and *B* in the population.

In the second model, the observations are made from a diploid individual, where there is an equal chance of the two observations originating from the same chromosome or from different chromosomes.

$$ {P}_1\left(A\wedge B\right)=\frac{1}{2}f(AB)+\frac{1}{2}f(A)f(B) $$

In the case where both observations are made from the same chromosome, the probability of observing alleles *A* and *B* is the frequency that *A* and *B* appear on the same chromosome in the population, i.e. the haplotype frequency, *f*(*AB*). Otherwise, the probability is the same as independently observing *A* and *B* on different chromosomes.

These two models are compared as a log-likelihood ratio, which is calculated as:

$$ \gamma \left(A,B\right)={ \log}_2\frac{P_1\left(A\wedge B\right)}{P_2\left(A\wedge B\right)} $$

Log-likelihood ratios are aggregated across the entire genome through summation of *γ* for pairs of SNPs in a set *S* of SNP pairs sampled from windows a set size across the genome.

$$ \Lambda (S) = {\displaystyle \sum_{\left(A,B\right)\in S}}\gamma \left(A,B\right) $$

This step can be repeated as a bootstrapping approach to estimate the empirical distribution of the genome-wide aggregated log-likelihood ratio.

#### Simulations

We performed coalescent simulations to test our method free of base errors and under various demographic scenarios. We used the coalescent simulator ms [35] to simulate diploid individuals and population reference panels of haplotypes for comparison. For each replicate, we simulated 3000 independent segments of 500 Kb in size for a total of 1.5 Gb. Segregating sites with minor allele frequencies lower than 10 % were removed. Reference panels consisted of 200 haplotypes. For diploid individuals, we simulated base observations from low-coverage sequencing by randomly drawing an allele from segregating sites at a rate of 0.01. This was done separately for each chromosome and sites where both alleles were observed were discarded, resulting in ~0.02 fold coverage. This process was repeated to construct multiple observation sets per individual.

The single simple population model used a constant effective population size of 10,000. The second model, representing the reference population and samples originating in distinct populations, simulated an ancestral population of 10,000 that split 100, 500, 1000, 2000, and 4000 generations ago into two equal sized populations of 10,000 each. The model of recent human history was based off of parameters inferred by Gutenkunst et al. [26] (see Additional file 1).

#### Reference panels

All human sequence and reference panel data used in this study were downloaded from public sources (accession details listed below). Institutional review and ethical approval were not required for this research.

We constructed reference panels of single nucleotide polymorphisms (SNPs) using the 1000 Genomes Project Phase one data set [25]. We filtered for biallelic SNPs that were polymorphic in the target population (CEU, GBR, etc) with a minimum minor allele count of 10. To avoid errors from mismapped reads, we restricted our panels to sites where all overlapping 35mers are unique across hg19 according to the Duke Uniq 35 track from the Mappability tracks on the UCSC Genome Browser [36].

#### Modern and ancient human sequence data

We obtained Illumina sequencing data from a European male (NA12891) and a European female (NA12892) sequenced as part of Platinum Genomes by Illumina, Inc [27] from the National Center for Biotechnology Information Sequence Read Archive (accession IDs ERR194160 and ERR194161) [37].

Illumina sequencing data from DNA extracted from 12 samples from 11 Bronze Age Eurasian humans [19] were downloaded from the European Nucleotide Archive (project accession ID PRJEB9021) [38]. We downloaded mapped reads in BAM format for samples RISE109, RISE154, RISE240, RISE247, RISE480, RISE483, RISE507, RISE508, RISE510, RISE546, RISE554, and RISE586.

### Availability of supporting data

Software written for this manuscript is available at http://github.com/svohr/tilde.

## Additional file

Additional file 1:
**Additional Methods and Results, Additional methods and additional simulation and sequencing results.** (PDF 532 kb)

## Abbreviations

STR: short tandem repeat
SNP: single nucleotide polymorphism
LD: linkage disequilibrium
LLR: log-likelihood ratio
