# Supplementary material for: A method for positive forensic identification of samples from extremely low-coverage sequence data
Source: BMC Genomics. 2015 Dec 7;16:1034. doi: 10.1186/s12864-015-2241-6 (PMC4672566; doi:10.1186/s12864-015-2241-6)
Supplement: Additional file 1: — Additional Methods and Results, Additional methods and additional simulation and sequencing results. (PDF 532 kb) [file 12864_2015_2241_MOESM1_ESM.pdf]

Additional Materials

Coalescent simulations

We performed coalescent simulations to test our method free of base errors and under various demographic scenarios. We used the coalescent simulator ms[1] to simulate diploid individuals and population reference panels of haplotypes for comparison. For each replicate, we simulated 3,000 independent segments of 500 Kb in size for a total of 1.5 Gb. Segregating sites with minor allele frequencies lower than 10% were removed. Reference panels consisted of 200 haplotypes. For diploid individuals, we simulated base observations from low-coverage sequencing by randomly drawn an allele from segregating sites at a rate of 0.01. This was done separately for each chromosome and sites where both alleles were observed were discarded, resulting in ~0.02 fold coverage. This process was repeated to construct multiple observation sets per individual.

The following ms commands were used to simulate samples from diploid individuals and reference panels of haplotypes.

Simulation 1: Single constant-size population

```
ms 204 3000 -t 200 -r 200 500000
```

Simulation 2: Individuals and reference panel drawn from different populations

Populations split 100 generations ago.

```
ms 204 3000 -t 200 -r 200 500000 -I 2 4 200 -ej 0.0025 1 2 -n 1 1 -n 2 1
```

Populations split 500 generations ago.

```
ms 204 3000 -t 200 -r 200 500000 -I 2 4 200 -ej 0.0125 1 2 -n 1 1 -n 2 1
```

Populations split 1,000 generations ago.

```
ms 204 3000 -t 200 -r 200 500000 -I 2 4 200 -ej 0.025 1 2 -n 1 1 -n 2 1
```

Populations split 2,000 generations ago.

```
ms 204 3000 -t 200 -r 200 500000 -I 2 4 200 -ej 0.05 1 2 -n 1 1 -n 2 1
```

Populations split 4,000 generations ago.

```
ms 204 3000 -t 200 -r 200 500000 -I 2 4 200 -ej 0.1 1 2 -n 1 1 -n 2 1
```

Simulation 3: Recent human demographic history

We tested our method on simulated data based on parameters inferred from allele frequency data [2]. This ms command below describes the population model for the 3 populations (Africa, Europe, Asia). We repeated the command for each population comparison, changing the number of chromosomes sampled from each population (highlighted in red).

```
ms 204 3000 -t 470 -r 200 500000 -I 3 204 0 0
     -n 1 1.23 -n 2 0.1 -n 3 0.051
     -en 0.0189 2 0.21 -en 0.1964 1 0.730
     -m 3 2 1.92 -m 2 3 1.92 -m 3 1 0.38 -m 1 3 0.38
     -m 2 1 0.6 -m 1 2 0.6
     -em 0.0189 2 1 5 -em 0.0189 1 2 5
     -ej 0.0189 3 2 -ej 0.125 2 1
```

In addition to the within population comparisons described in the paper, we also compared samples from diploid individuals from one population using a reference panel drawn from another population. We found that power to distinguish between different individuals is diminished when the reference panel drawn from a distantly related population.

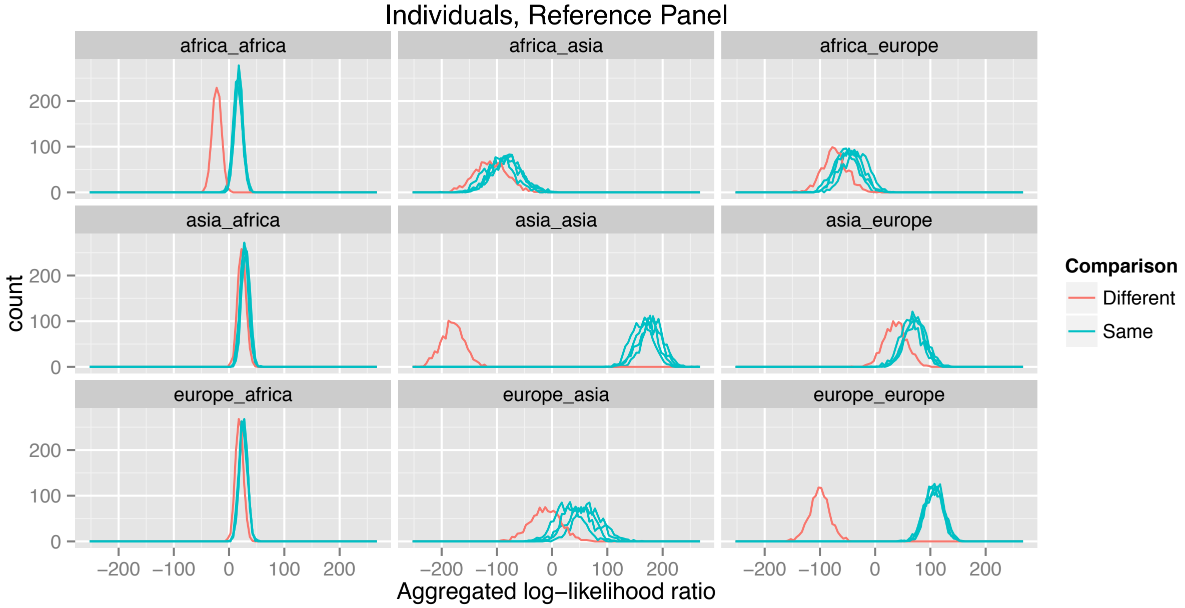

Figure S1: Comparisons between samples from diploid individuals using different reference panels (window labels indicate populations of "Individuals_ReferencePanel")

Simulation 4: Related individuals

We tested the utility of our method to detect close familiar relationships between individuals using low-coverage samples. Under the same population model as Simulation 1, we simulated diploid individuals that would be "parents" for two child individuals. We generated 2 children by drawing 1 chromosome at random from each diploid parent for

each simulated segment. This produced two children that shared ~50% of the genome with each other and with their parents. Allele observations were sampled from the diploid children using the same strategy as before. We compared the two children with samples from one parent, and a third, unrelated diploid individual drawn directly from the simulated haplotypes. As expected, the aggregated log-likelihood values for the parent-child and sibling-sibling comparisons fall directly between the same individual comparisons and the unrelated individual comparisons (Figure S1).

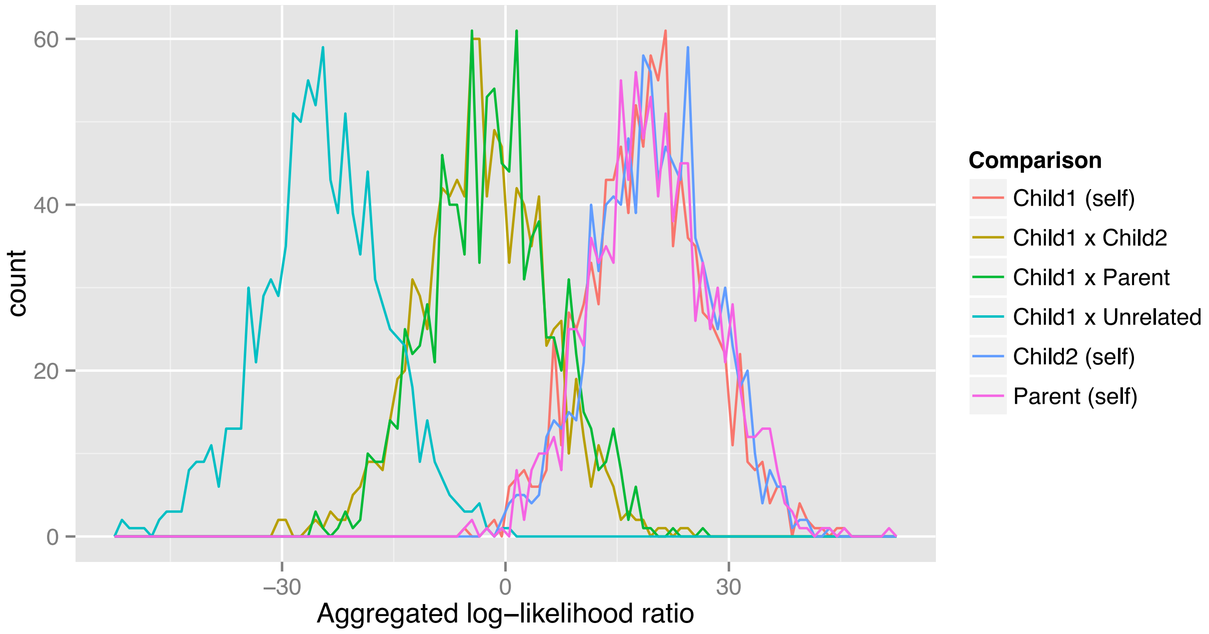

Figure S2: Results of comparisons of simulated first-order relatives are distinct when compared with results from unrelated individuals and the same individual.

Experiments with Sequencing Data

We constructed reference panels of single nucleotide polymorphisms (SNPs) using the 1000 Genomes Project Phase 1 data set[3]. We filtered for biallelic SNPs that were polymorphic in the target population with a minimum minor allele count of 10. To avoid errors from mismapped reads, we restricted our panels to sites where all overlapping 35mers are unique across hg19 according to the Duke Uniq 35 track from the Mapability tracks on the UCSC Genome Browser. We constructed 7 reference panels in total; 5 from Europe (CEU, FIN, GBR, IBS, and TSI), 1 from Asia (CHB) and 1 from Africa (YRI).

We obtained Illumina sequencing data from a European male (NA12891) and a European female (NA12892) sequenced as part of Platinum Genomes by Illumina, Inc (http://www.illumina.com/platinumgenomes/) from the National Center for Biotechnology Information Sequence Read Archive (http://www.ncbi.nlm.nih.gov/sra/, accession ERR194160 and ERR194161). We sampled 3,200, 32,000, 160,000, 320,000 and 1,600,000 reads without replacement to approximate 0.01%, 0.1%, 0.5%, 1.0%, and 5.0% x

coverage of the nuclear genome to generate two sets at each coverage level for both individuals. Reads were mapped to the human reference sequence (GRCh37/hg19) using bwa mem [4] with default parameters.

Single base observations were made from mapped reads using output from samtools mpileup. Reads were required to have a map quality greater than 30 and bases were required to have a base quality of 30. Positions where more than one read mapped were excluded.

The comparison describe in the main text was repeated with the substitution of the CEU reference panel with alternatives (FIN, GBR, IBS, TSI, CHB, YRI). We found that all European populations produced similar results.

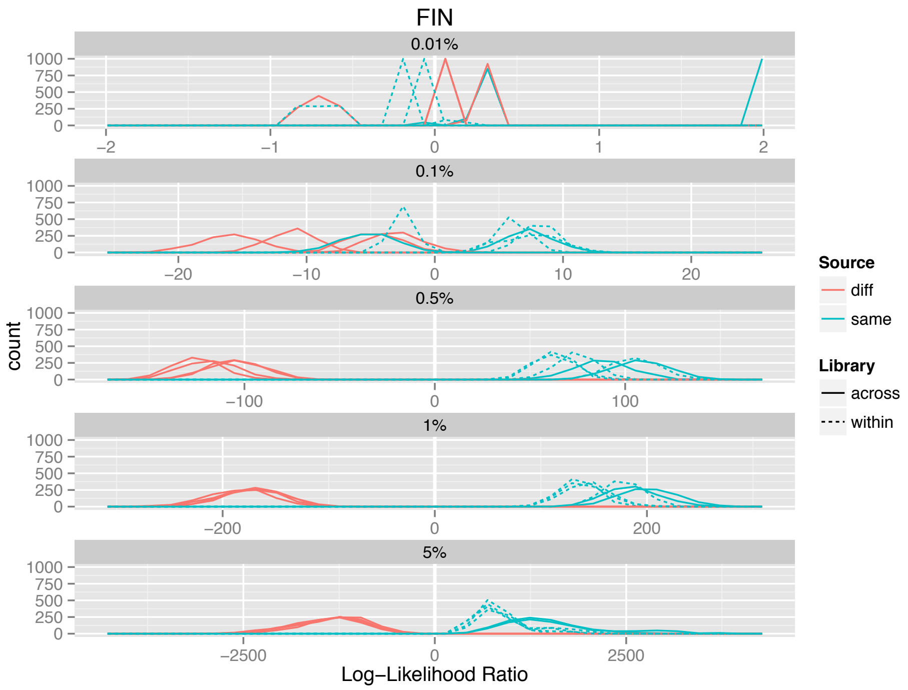

Figure S3: Results from low-coverage sequencing comparison using Finnish in Finland (FIN) population as the reference panel.

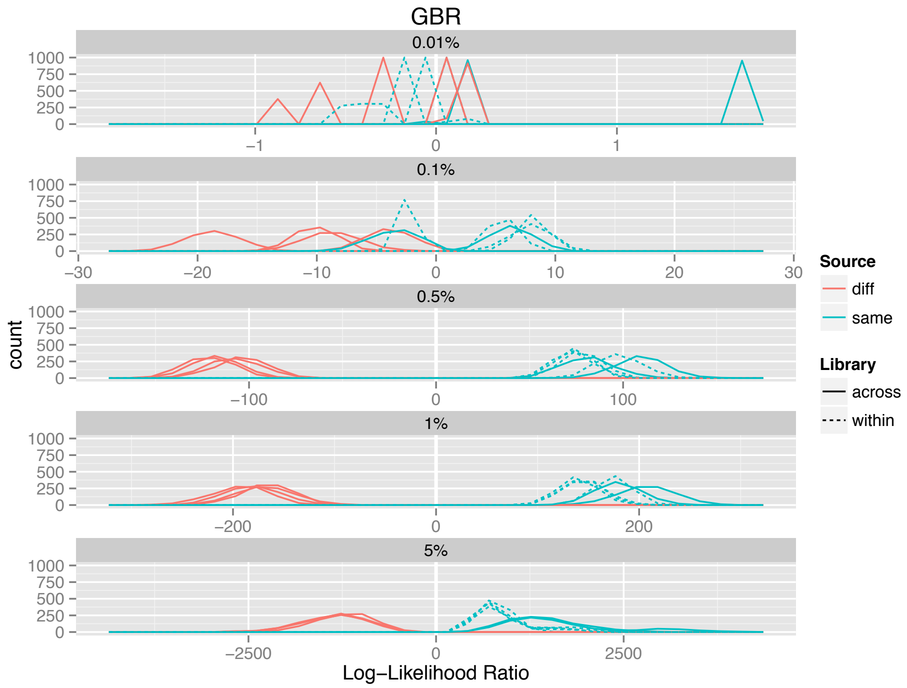

Figure S4: Results from low-coverage sequencing comparison using British in England and Scotland (GBR) population as the reference panel.

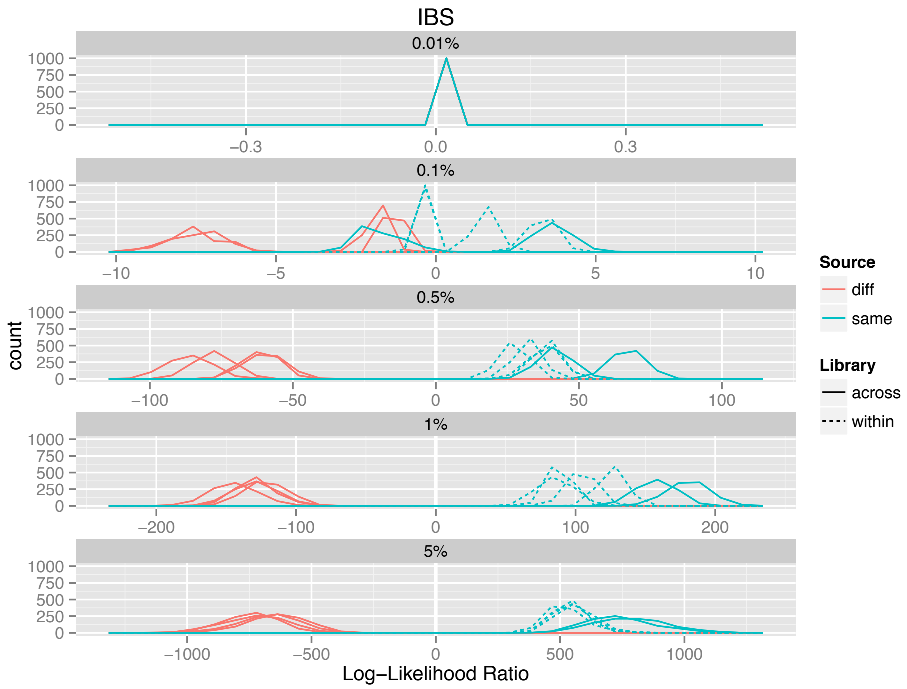

Figure S5: Results from low-coverage sequencing comparison using Iberian populations in Spain (IBS) population as the reference panel.

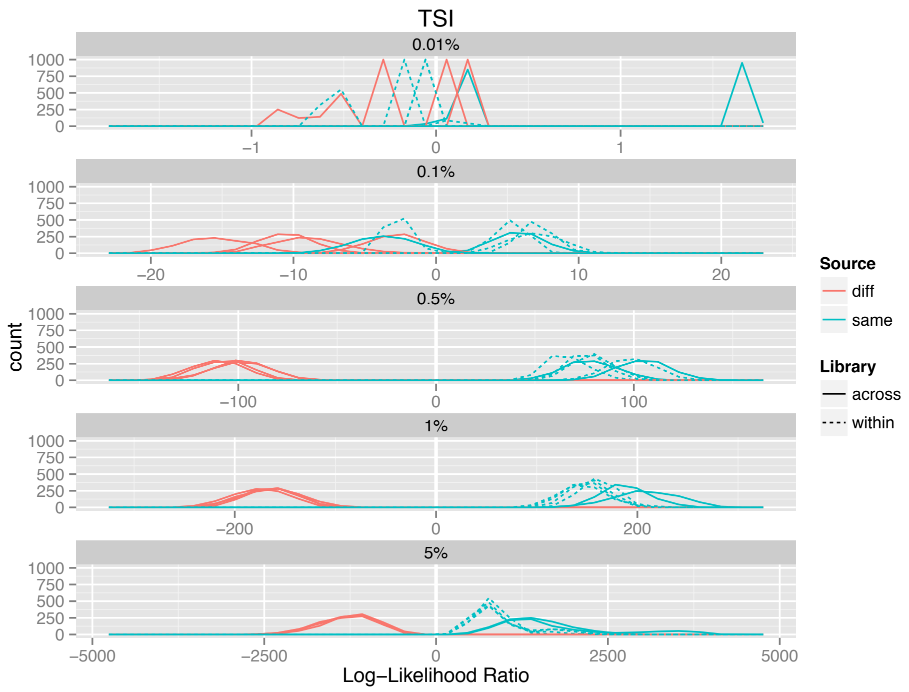

Figure S6: Results from low-coverage sequencing comparison using Toscani in Italia (TSI) population as the reference panel.

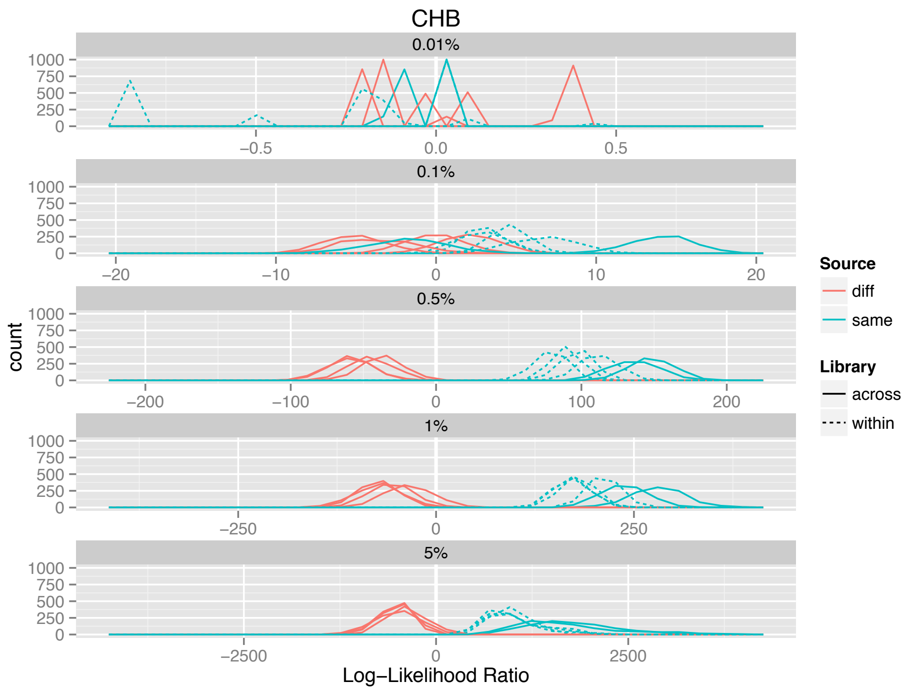

Figure S7: Results from low-coverage sequencing comparison using Han Chinese in Beijing, China (CHB) population as the reference panel.

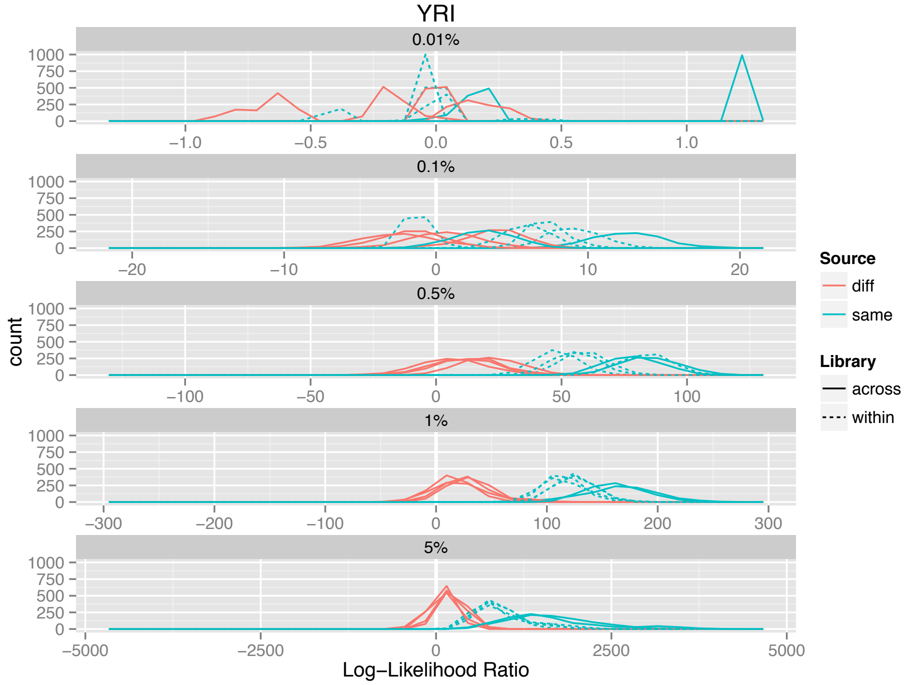

Figure S8: Results from low-coverage sequencing comparison using Yoruba in Ibadan, Nigeria (YRI) population as the reference panel.

Comparisons were also made across coverage levels for the original CEU reference panel. This demonstrates that comparisons with a sample with extremely low coverage can benefit higher coverage in the other sample.

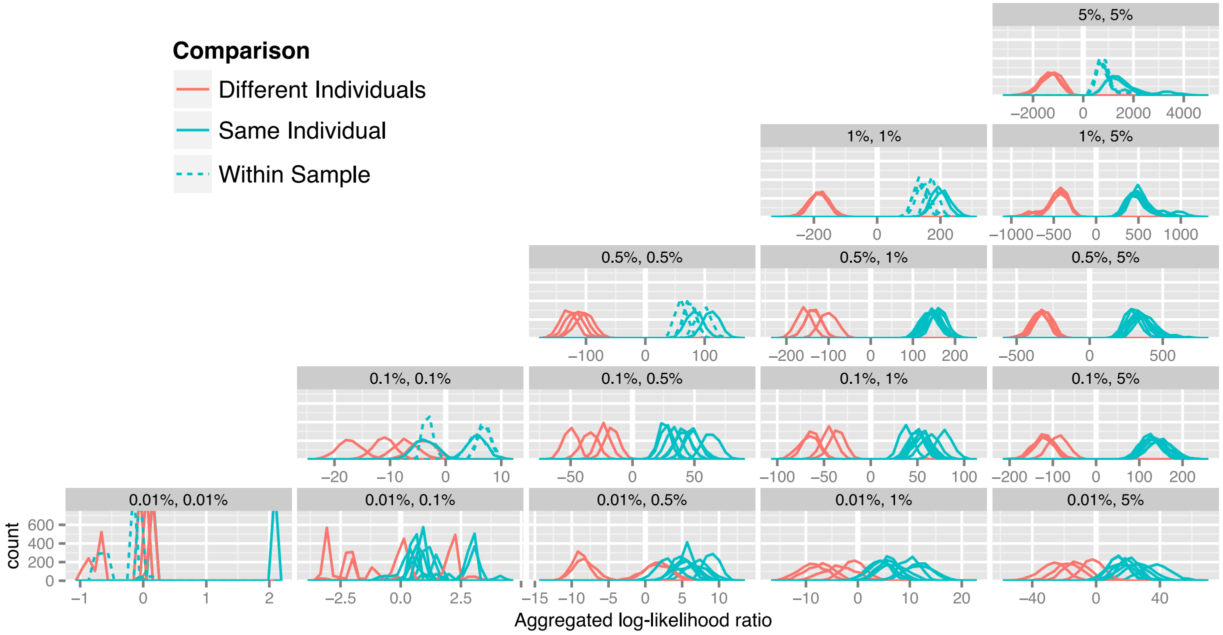

Figure S9: Comparisons of samples with different levels of coverage using CEU reference panel.

Ancient DNA

Illumina sequencing data from DNA extracted from 12 samples from 11 Bronze Age Eurasian humans [5] were downloaded from the European Nucleotide Archive (project accession number PRJEB9021). We downloaded mapped reads in BAM format for samples RISE109, RISE154, RISE240, RISE247, RISE480, RISE483, RISE507, RISE508, RISE510, RISE546, RISE554, and RISE586.

Again, single base observations were made from mapped reads using output from samtools mpileup [6]. Reads were required to have a map quality greater than 30 and bases were required to have a base quality of 30. Positions where more than one read mapped were excluded. In addition, only mutations that represented transversion mutations were included in the analysis.

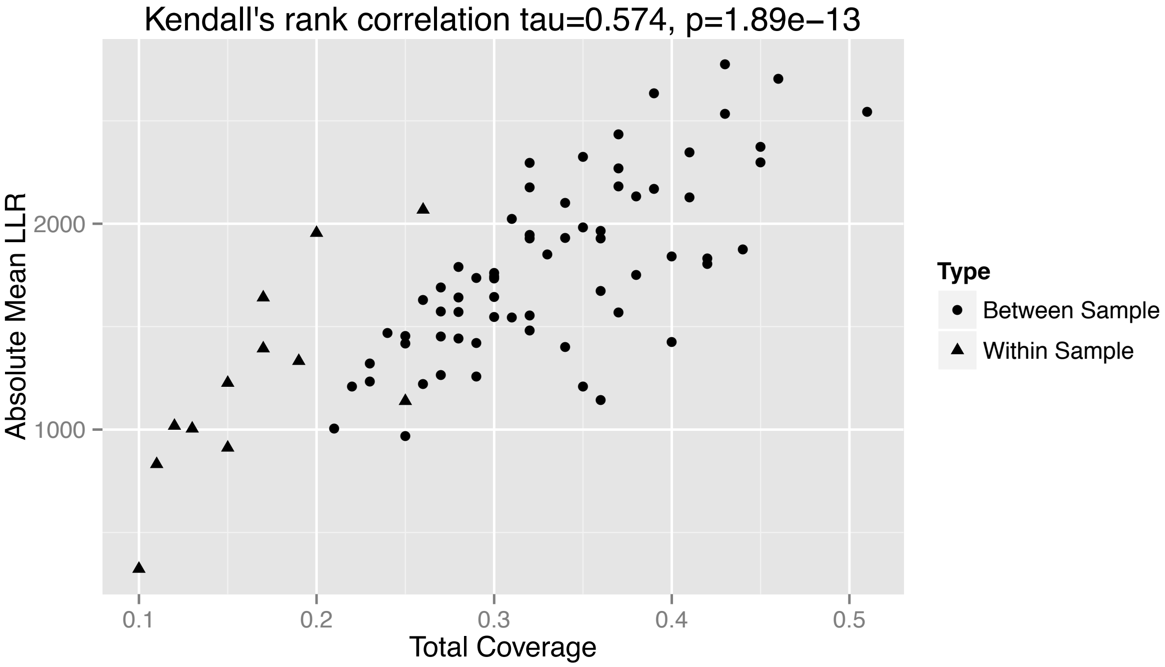

Figure S10: From ancient DNA samples, total sequence coverage in comparison is correlated with the absolute value of the mean aggregated log-likelihood ratio.

References

1. Hudson RR: **Generating samples under a Wright-Fisher neutral model of genetic variation.** *Bioinformatics* 2002, **18**:337–338.

2. Gutenkunst RN, Hernandez RD, Williamson SH, Bustamante CD: **Inferring the joint demographic history of multiple populations from multidimensional SNP frequency data.** *PLoS Genet* 2009, **5**:e1000695.

3. Abecasis GR, Auton A, Brooks LD, DePristo M a, Durbin RM, Handsaker RE, Kang HM, Marth GT, McVean G a: **An integrated map of genetic variation from 1,092 human genomes.** *Nature* 2012, **491**:56–65.

4. Li H: **Aligning sequence reads, clone sequences and assembly contigs with BWA-MEM**. *arXiv Prepr arXiv* 2013, **00**:3.

5. Allentoft ME, Sikora M, Sjögren K-G, Rasmussen S, Rasmussen M, Stenderup J, Damgaard PB, Schroeder H, Ahlström T, Vinner L, Malaspinas A-S, Margaryan A, Higham T, Chivall D, Lynnerup N, Harvig L, Baron J, Casa P Della, Dąbrowski P, Duffy PR, Ebel A V., Epimakhov A, Frei K, Furmanek M, Gralak T, Gromov A, Gronkiewicz S, Grupe G, Hajdu T, Jarysz R, et al.: **Population genomics of Bronze Age Eurasia**. *Nature* 2015, **522**:167–172.

6. Li H, Handsaker B, Wysoker A, Fennell T, Ruan J, Homer N, Marth G, Abecasis G, Durbin R: **The Sequence Alignment/Map format and SAMtools**. *Bioinformatics* 2009, **25**:2078–2079.
